# Predicting User Susceptibility to Phishing Based on Multidimensional Features

**DOI:** 10.1155/2022/7058972

**Published:** 2022-01-17

**Authors:** Rundong Yang, Kangfeng Zheng, Bin Wu, Di Li, Zhe Wang, Xiujuan Wang

**Affiliations:** ^1^School of Cyberspace Security, Beijing University of Posts and Telecommunications, Beijing 100876, China; ^2^School of Computer Science, Beijing University of Technology, Beijing 100124, China

## Abstract

While antiphishing techniques have evolved over the years, phishing remains one of the most threatening attacks on current network security. This is because phishing exploits one of the weakest links in a network system—people. The purpose of this research is to predict the possible phishing victims. In this study, we propose the multidimensional phishing susceptibility prediction model (MPSPM) to implement the prediction of user phishing susceptibility. We constructed two types of emails: legitimate emails and phishing emails. We gathered 1105 volunteers to join our experiment by recruiting volunteers. We sent these emails to volunteers and collected their demographic, personality, knowledge experience, security behavior, and cognitive processes by means of a questionnaire. We then applied 7 supervised learning methods to classify these volunteers into two categories using multidimensional features: susceptible and nonsusceptible. The experimental results indicated that some machine learning methods have high accuracy in predicting user phishing susceptibility, with a maximum accuracy rate of 89.04%. We conclude our study with a discussion of our findings and their future implications.

## 1. Introduction

Phishing mainly uses social engineering and technical deception to obtain private user information. The most typical phishing attack lures the recipient to a phishing website that is carefully designed to closely resemble the target organization's website and obtains sensitive personal information entered by the recipient [1].

Recently, with the rapid development of the communication industry and Internet-related technologies, people are using the Internet more frequently, and online activities are increasing. While people enjoy the convenience brought by the Internet, phishing and other attacks are threatening their lives. The areas related to users' property are the most affected by phishing attacks, which seriously affect the safety of people's online transactions. Phishing has become one of the most threatening and widely used network attacks owing to its rapid development, potential to cause huge losses, and serious hazards.

Phishing attacks affect more than 40 million Internet users each year. According to an APWG (Antiphishing Working Group) report [2], 15208832 phishing websites and 103347 phishing emails were detected in 2020. Furthermore, phishing activities peaked in October 2020, and 369254 phishing attacks occurred in January 2020 alone. The frequency of phishing attacks continues to grow, and the property securities of Internet users, businesses, and organizations remain at great risk. According to an FBI report, $26 billion was lost globally to business email compromise or email account compromise (BEC/EAC) crimes from June 2016 to July 2019. Studies report that Internet users perform poorly in distinguishing legitimate websites from phishing sites; users are unable to correctly identify phishing sites 40–80% of the time, and 70% of users are willing to transact with phishing sites [3].

To prevent and identify phishing, researchers have proposed many antiphishing detection techniques, mainly using technologies for defense, such as black and white list-based, heuristic-based, machine learning-based, and deep learning-based detection techniques. Although there are many antiphishing detection techniques, the success rate of phishing attacks remains high. Experts agree that phishing remains a crucial problem that has not yet been solved. This is because phishing attacks are aimed at people rather than machines. Phishing attackers try to exploit the susceptibility of users to achieve malicious goals.

There exists vast literature on the analysis of factors influencing phishing susceptibility that aim to explore which factors cause people to be more susceptible to deception. These studies all aim to design antiphishing techniques to curb phishing, but there is little current research and no consensus on predicting which users are more likely to be phished. For phishing susceptibility models, previous studies have focused on single user characteristics, such as personality, cognitive processes, and demographic information. However, the factors that influence a person's susceptibility to phishing are the result of multiple characteristics acting simultaneously. Therefore, there is a need to combine the factors that influence susceptibility for a more comprehensive analysis. The main research objectives of this study are as follows: examining the factors that influence human susceptibility, assessing the effectiveness of phishing, and predicting the susceptibility of users to phishing.

The main contributions of our research can be summarized as follows:

1. In this study, an MPSPM model is designed to predict the susceptibility to phishing
2. Our research validates existing theories and contributes to the discovery of factors that affect the network susceptibility
3. Our research improves the interpretability of the theory and analyzes the factors that have the greatest impact on susceptibility to phishing

To study the phishing susceptibility problem, we administered different phishing attacks to recruit volunteers and collected data on their demographic information, personality, security knowledge, and overall awareness using a questionnaire; then, we predicted their susceptibility using 7 different supervised machine learning methods with high prediction accuracy. We used the most accurate prediction model to provide an in-depth analysis of the factors influencing user susceptibility.

The remainder of the study is organized as follows: Section 2 introduces certain problems related to phishing susceptibility analysis.  Section 3 introduces the MPSPM model for phishing susceptibility analysis and an analysis of factors included in phishing susceptibility analysis.  Section 4 provides details of data collection and feature extraction of susceptibility impact factors.  Section 5 analyzes the experimental results of the proposed method.  Section 6 draws conclusions based on our findings and provides details regarding future research directions.

## 2. Related Research

Currently, most of the research on antiphishing focuses on testing antiphishing tools and antiphishing algorithms [1, 3], and some research focuses on improving the detection of phishing [4, 5]. These current studies can accurately detect phishing, but the number of annual phishing victims remains high, which indicates that phishing attacks people and not systems. Therefore, an increasing number of researchers are shifting their focus to the analysis of user susceptibility. Phishing susceptibility is the degree to which users interact with phishing attacks [6]. Over time, researchers have made some progress and proposed various phishing susceptibility models that explain or describe the factors behind phishing susceptibility [7, 8].

According to Alnajim and Munro [9], the quantitative factors with the greatest impact on user susceptibility to phishing are phishing awareness and technical ability. When validating their model, the experimental result demonstrated that the user awareness is a key factor in distinguishing between phishing and legitimate sites.

Parrish et al. [10] designed a phishing susceptibility framework (PFS) in which the main factors considered to influence phishing susceptibility were demographics, experience, Big Five personality, and threat type.

Sheng et al. [11] used a questionnaire to collect demographics, risk propensity, and phishing knowledge information to analyze their influence on phishing and proposed a model called DRKM. The collected demographic information mainly included age, gender, and education. The risk propensity is calculated as the willingness to participate in risky behaviors. The knowledge and experience are mainly described as the awareness of phishing and ability to rely on the Internet. Their experimental results indicated that gender, age, and risk propensity were closely related to the prediction of phishing susceptibility.

Wang et al. [12] designed a phishing susceptibility model (PSM) that mainly focused on analyzing the susceptibility to phishing emails, where the main analysis factor was the threat. Based on the experimental results, they found that the key factors affecting phishing email susceptibility were knowledge, deception indicators, and potential cues. The PFM model combines elements of other models while adding multiple new influencing factors via a multistage fusion feature approach to analyze the factors influencing phishing susceptibility.

Although various phishing susceptibility models have been proposed, no definitive conclusion has been reached regarding the susceptibility factors. Current studies have analyzed the causes of phishing susceptibility and explained the main principles of phishing, many of which are based on psychological and statistical analyses. Very few studies have analyzed the potential phishing victims. Currently, studies mostly analyze demographic characteristics, but there are no definitive conclusions.

In the literatures [13, 14], it was found that age and gender do not play a role in susceptibility to phishing. However, in some studies, such as [15, 16], women were deemed more likely to trust Internet connection compared to men, a situation that is also influenced by the environment. In addition, studies [11, 17] concluded that women are mostly phishing targets with a higher success rate. However, the conclusions of these studies have not been validated because they were mainly conducted on college students. Consequently, we can draw a vital conclusion: personal characteristics do not majorly influence phishing susceptibility. Other studies have examined other characteristics, such as the financial data, Internet characteristics, and length of time online [13], but these characteristics do not have much impact on phishing susceptibility either. Some studies have addressed other personal characteristics [18–21]. For example, it has been determined that trust is a major factor influencing phishing susceptibility, which is supported by the fact that spear phishing has a higher attack success rate than that of regular phishing because spear phishing often uses fear and urgency to threaten the victim and make them vulnerable to phishing. However, these influences can be mitigated by security awareness training. The literature [22] focuses more on the human impact on the phishing emails.

Steves et al. [23] built the previous research to construct a phishing scale, which is a phishing tool that utilizes premise to its information retrieval. The former analyzes the user's area of interest in phishing messages, and the latter indicates the attack elements contained in phishing attacks. According to experiments, phishing success is high when the former agrees heavily with the latter.

The literature [6] considers demographic information as an important factor of phishing susceptibility and argues that demographic characteristics contain human vulnerability, which is the focus of research.

There are many current studies on the susceptibility to phishing, particularly, one based on psychology and statistical learning and another based on machine learning. We compare and analyze the differences between statistical learning and machine learning. Statistical learning focuses on statistical analysis of individuals and inferring the behavior of groups, whereas machine learning is focused on batch data training to make predictions about individuals without an in-depth understanding of their action mechanisms. One of the goals of our study is to predict online phishing susceptibility; hence, we believe that using machine learning is more suited to achieving our goals. We obtained data by means of a questionnaire, preprocessed the data, and used a machine learning modeling approach to predict phishing susceptibility; then, we used the prediction results to strengthen the theory and have a positive practical impact.

Because previous studies either did not reach conclusions or lacked some data features that could be used as influencing factors of phishing susceptibility, we tried to obtain data directly by means of questionnaires and derive models directly from the data instead of using the relational conclusions of previous studies, especially because many previous studies contradict with each other. The goal of our study is to investigate the vulnerability of users to phishing according to their characteristics. Phishing can be better avoided by intervening with the actions of those who are more vulnerable to it. This approach is top-down and does not require a direct interpretation of the correctness of the theory and makes our research more meaningful.

Regarding the data at the hand, there may be relationships that have not been discovered or are difficult to hypothesize; new relationships may be discovered using machine learning predictive models to facilitate the development of theories on susceptibility influences. By analyzing the characteristics of previous influences, existing models can be improved for interpretability, and existing theories can be validated to prevent theories to diverge from reality.

MPSPM combines elements of each existing model and introduces new elements in terms of incorporated independent variables, integration of multiple decision stages, and parsimonious model estimation that consider user heterogeneity to predict susceptibility.

## 3. Proposed Method

**Figure 1:**
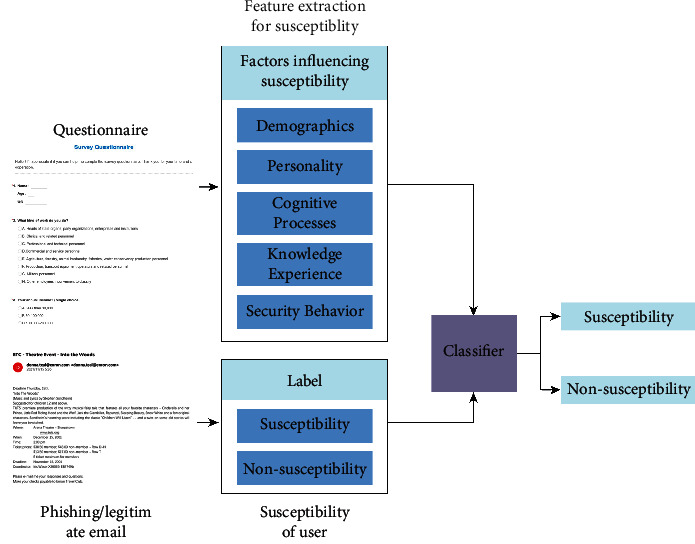
A phishing susceptibility prediction model. The model contains a susceptibility feature extraction part, a classification part, and a model prediction part, which enables prediction of susceptibility.

Here, we recruited a total of 1105 volunteers to participate in this study. The phishing susceptibility model (MPSPM) designed in this study is shown in Figure 1. The MPSPM model is mainly used for phishing susceptibility prediction and mainly considers 5 categories of decision factors that affect the susceptibility related to phishing sites, including demographics, personality, cognitive processes, knowledge and experience, and security behavior. These are used as features for predicting user susceptibility using multidimensional features and multiple supervised machine learning methods for prediction. The rest of the section details the susceptibility factors and model specifics.

## 4. Data Collection and Feature Extraction of Susceptibility Impact Factors

The MPSPM model, by using questionnaire information on all susceptibility influencing factors, data were collected, which are defined as the influencing factors that were included in the MPSPM model, mainly considered the following aspects: demographics, personality, cognitive process, knowledge and experience, and security behavior. Because there is no single theoretical framework to support these factors, we mainly consulted the following theories: PMT [24], heuristic systems model [25], STPS [26], security behavior scale [27], and FFM [28]. The factors considered are presented as follows.

### 4.1. Demographic Factors

Demographic characteristics have been studied in many studies as important factors influencing phishing susceptibility. Gender is a very important factor, and the literature [29] demonstrated that men are more concerned with the instrumental outcomes, while women are more concerned with the process. The literatures [10, 11] also suggest that gender has a greater impact on phishing susceptibility.

Age is important in influencing the susceptibility to phishing as well. The literature [29] demonstrated that age has an important influence on the use of technology. In addition, studies on the age factor [10, 30] have confirmed the importance of age in phishing susceptibility. Similarly, previous studies have demonstrated that education has a differential impact on adoption and use of technology [31]. In the context of phishing, education may be related to technical training and knowledge, which may influence phishing susceptibility.

### 4.2. Personality Factors

The relationship between FFM personality and the principle of persuasiveness is discussed in the literature [28] regarding the analysis of the Big Five's susceptibility to phishing. The “Social Engineering Personality Framework” theory was proposed, and the influence of the Big Five personality traits on phishing susceptibility was explained based on previous research. The following conclusions were drawn.

  Openness: defines a higher susceptibility to liking, scarcity, and social proof because extroverts also have a higher association with sociality
  Conscientiousness: refers to a higher susceptibility to authority, commitment consistency, and reciprocity because people with this personality trait blindly exchange compliance with rules
  Agreeableness: indicates a susceptibility to all persuasiveness but greater vulnerability to persuasive principles, such as social proof, authority, reciprocity, and liking, because people with this personality trait are particularly trusting of others
  Extraversion: susceptibility is high for this personality type, but some studies have shown that the susceptibility to phishing of extraverted people is highly correlated with computer proficiency; higher proficiency resulted in having lower susceptibility.
  Neuroticism: neuroticism was associated with increased computer anxiety and security-related retention, which reduced susceptibility; however, the authors concluded that individuals scoring higher on this trait may be susceptible to the principle of authority because of their tendency to comply with instructions and orders from authorities.

Although the original authors propose a theoretical framework, these hypotheses need further validation. Consequently, the proposed framework remains a theoretical approach to be implemented.

### 4.3. Cognitive Processes Factors

Cognitive processes: the mental processes by which individuals acquire knowledge and understanding. Through these processes, the human cognition is formed, which includes recognition, thinking, judgment, and memory [32]. The literature [33] proposed a theoretical framework to study the factors of phishing susceptibility, proposed the susceptibility of information processing to phishing emails, and explained how phishing emails affect human susceptibility. Information processing is an important aspect of cognition, through which people process and judge external stimuli to obtain information [25]. Among the main models on information processing are the HSM, elaboration likelihood model (ELM), and social judgment theory (SJT) [34]. Many studies have shown that the HSM framework is very effective for studying phishing susceptibility and can analyze possible influences on phishing [35–37]. On the other hand, a systematic approach provides a deeper way of processing information that requires more cognitive effort. People prefer to analyze information and usually tend to do their own research to assess the credibility of the received information [38].

### 4.4. Knowledge and Experiences Factors

Knowledge and experience are the important influencing factors of phishing risk, and many studies have found that knowledge and experience have a great influence on whether phishing attacks are successful or not. The knowledge about phishing mainly comprises computer knowledge, network security knowledge, network usage experience, and social engineering-related knowledge. In the literature [7], it is expressed that those with a high level of knowledge have a lower risk of being phished and are less likely to click on links when faced with phishing emails. The findings of study [25] indicated that those who were knowledgeable about emails responded to fewer emails, and the authors concluded that knowledge and experience would better help people distinguish between legitimate and phishing emails. Vishwanath et al. [39] found that knowledge and experience would also influence the attention and evaluation of email content, which influenced the subjects' decision-making behavior.

In real life, many victims of phishing have a low security awareness and lack an understanding of online fraud. Knowledge and experience can help users understand security warnings and better avoid potential risks. Thus, knowledgeable and experienced users are less likely to be deceived. Consequently, increasing users' knowledge and experience can be an important intervention tool to reduce phishing risks.

### 4.5. Security Behavior Factors

The user's security behavior has a relatively high correlation with susceptibility to phishing; to analyze the intention related to assessing the user's security behavior, Downs et al. [7] designed a security behavior scale (SeBIS). This scale consists of 16 items that are categorized into the following:

- Password generation (e.g., strong passwords and use of password management tools)
- System updates
- Device security (i.e., locking devices)
- Proactive awareness (i.e., considering and acting on security alerts)

In the literature, the authors analyzed the correlation between the SeBIS and other psychometric tools. The results showed that the correlation between DoSpeRT and SeBIS was positive, but for SeBIS and GDMS, both negative and positive correlations were observed between the tendency to procrastinate and rational style of decision-making, respectively.

**Figure 2:**
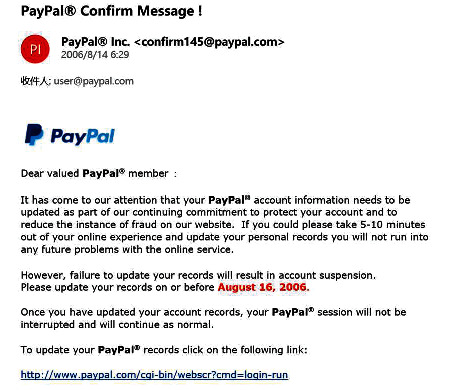
Phishing email. This is a fake PayPal phishing email.

Table 1 provides a summary of all data related to the factors included in the analysis, except for information about location and name to ensure anonymity. Occupations with low representation were classified as “others.” We constructed 3 legitimate and 4 phishing emails containing Bitcoin rewards and PayPal account hazards, as shown in Figure 2, after collecting and introducing all the statistics that had an impact on susceptibility into the susceptibility prediction model.

### 4.6. Model Predictions

The purpose of this study is to find an accurate method that can effectively predict people with high phishing susceptibility and conduct an in-depth analysis of people with high phishing susceptibility to find appropriate intervention methods to stop phishing in a timely and effective manner. The prediction model is used to determine whether there are common characteristics of people with high phishing susceptibility.

First, a set of 7 emails comprising 3 legitimate and 4 phishing emails were sent to all volunteers. After reviewing the emails, the volunteers were asked the following questions through a questionnaire: “Are they phishing emails?” and “Would you click on the links in the emails?” According to their assessment of phishing emails, the volunteers were divided into two categories: those who were easily deceived and those who were not easily deceived by phishing.

In this study, we analyze current supervised machine learning algorithms to select a machine learning model that is suitable to this topic, and we use the collected data to evaluate the correctness of the model. Our criteria for selecting supervised machine learning models are focused on how much the models are suitable for classification. We selected the following machine learning models: logistic regression (LR), gradient boosting decision tree (GBDT), support vector machine (SVM), decision tree (DT), random forest (RF), eXtreme gradient boosting (XGBoost), and adaptive boosting (AdaBoost) [39, 40].

Finally, we analyzed the prediction power of these machine learning algorithms by using them on the statistical data of the factors influencing the susceptibility to phishing obtained using a questionnaire.

## 5. Results and Discussion

The phishing experiment included two types of emails—legitimate and phishing emails—sent to 1105 volunteers. Table 1 provides the list of the people who were successfully attacked, which amounts to a total of 609 people; those are people with high susceptibility to phishing; roughly 55.11% were successfully attacked; this number is slightly higher than the average expected percentage.

We only used the questionnaire to determine whether the respondents would click on the fake links one-by-one; we do not ask respondents to take further action. Overall, the click-through rate suggests that phishing is a serious threat and should receive a great deal of attention considering the greater damage it can cause. Furthermore, our findings confirm that certain cues contained in the messages inhibit a more systematic processing of their content because phishing messages stimulate different people differently, which underlines the deceptive nature of phishing emails.

### 5.1. Experiment 1 Evaluating MPSPM Model Performance

To build the phishing susceptibility prediction model, we categorized the phishing victims into a high phishing susceptibility category, referred simply as phishing. This was done primarily because the validity of any potential findings for that group only, using this classification, is worth considering. Second, for most phishing training programs, all phishings are grouped into one category for analysis; hence, it is sufficient to know who the potential victims are. We identified that 609 of the 1105 volunteers were scammed. In other words, almost 55% of the volunteers were affected by scam messages. This means that these abducted individuals still represent the number of deceived individuals.

To validate our model, we implemented *k*-fold cross-validation (*k* = 10) by dividing the data into *k* subsets of equal sizes (stratified sampling). The validation process was then run *k* times, where k−1 subsets were used for model training and one subset was used for testing (different each time).

**Figure 3:**
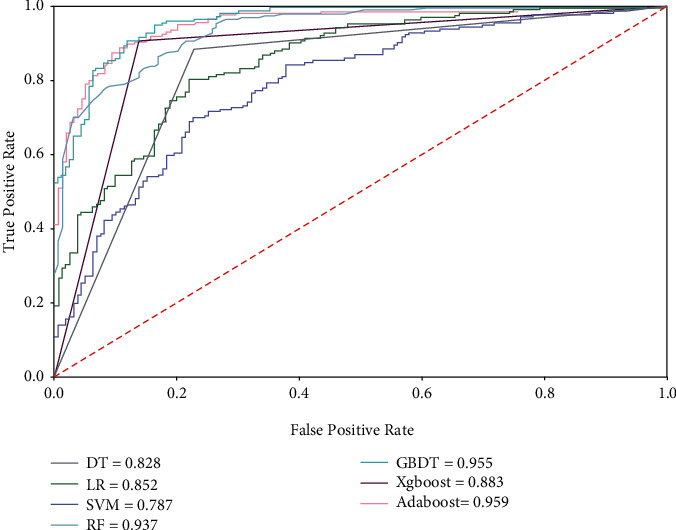
ROC curves of predictions across models and methods. The figure includes the ROC curves of several models for comparison.

Given that the MPSPM model is a categorical analysis tool, to receive more information, we use the characteristic (ROC) curve and area under the curve (AUC) values to evaluate the trade-off between true/false positives of the prediction model. As shown in Figure 3, notice that the GBDT, XGBC, and GBM models have a clear advantage, with true positive rates of 5–17% higher compared to the LR, TR, SVM, and RF models. In Figure 3, the accompanying ROC curves depict the trade-off between true (*y*-axis) and false (*x*-axis) positive rates for the models, with GBDT and GBM models outperforming their counterparts in terms of false positive rates at most levels of the ROC curves and having significantly higher true positive rates. The best overall model is the GBTD method.

**Figure 4:**
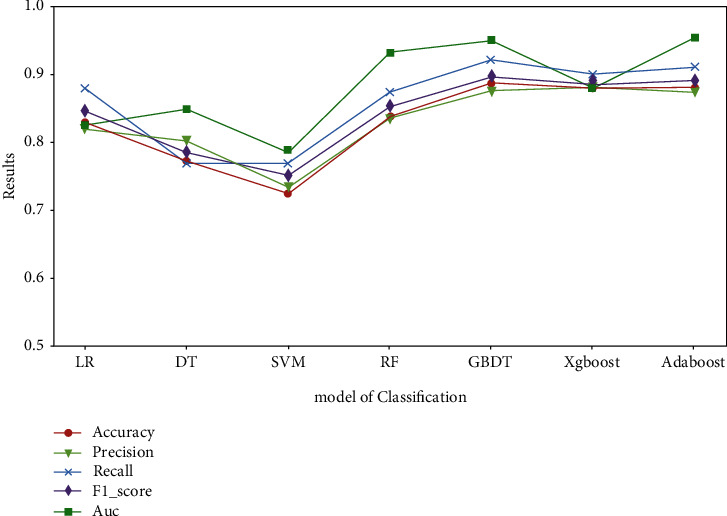
Scores regarding classification models. Comparison results of each learning algorithm used in the prediction model for each dataset.

Table 2 provides a summary of the binary classification models run using the selected supervised learning algorithms. Multiple dimensional attributes were used to be predicted as binary outcomes (phished or unphished). For each method, the following performance metrics were computed: accuracy (ACC), precision, recall, and F1-score. To more clearly represent the analysis of the results for each algorithm, Figure 4 shows a graph of the performance metrics for the different models, and it can be seen that the GBDT algorithm is the best in several dimensions: accuracy (ACC), precision, recall, and F1-score.

In terms of ACC, GBDT correctly predicted 89.04% of the cases, followed by AdaBoost and XGBoost (88.46% and 88.47%, respectively) and RF (84.14%). The remaining three algorithms, DT, LR, and SVM, achieved lower ACC scores in the 80% range and consistently scored lower in all other model evaluation metrics. Consistent with the ACC scores, GBDT had the highest recall (92.55%), followed closely by XGBoost, AdaBoost, RF, and DT. In contrast, SVM and LR had lower recall scores, reflecting their overall poor performance. The scores related to the other metrics are consistent with the abovementioned scores, with the largest differences occurring in the model precision ratings. On average, GBDT had the highest overall score, followed by XGBoost, AdaBoost, and DT.

### 5.2. Experiment 2 Analysis of Factors Influencing Susceptibility to Phishing

Next, we assess the subsequent goal of our study to discover more details about the potential victims. First, we wanted to know if there were any similarities between the different classification methods used in our study, especially considering that the ACC measures of these methods sometimes differed significantly from each other.

**Figure 5:**
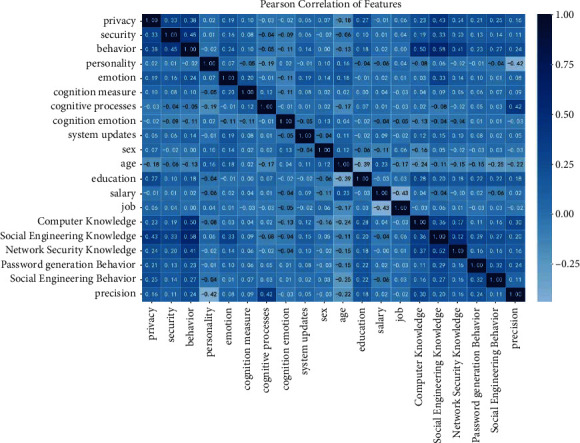
Correlation analysis was performed to optimize the model..

For this reason, we optimized the attributes in each model to maximize the prediction of the variable named being phished and set it equal to “Y” to determine which attribute values were most influential in predicting the outcome of a specified category. The local importance of an attribute is calculated based on its correlation with the predictions of its neighbors, which is maximized. The correlation matrix is shown in Figure 5.

To investigate which factors have the greatest impact on phishing, we turned our attention to the GBDT model for two reasons: first, it is the best performing model, and second, it is simpler compared to the others.

**Figure 6:**
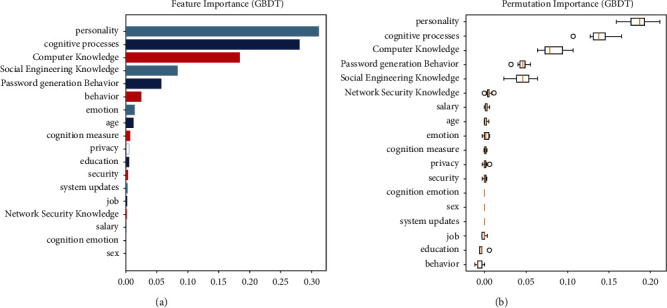
The influence of personality determined by analyzing the factors influencing the susceptibility to phishing and identifying the most influential factors.

We analyzed the GBDT model using feature importance. The analysis results are shown in Figure 6. We found that personality contributes the most to the model, indicating that personality is the most important factor influencing the susceptibility to phishing. This shows that, despite being knowledgeable and experienced, when people encounter something new, their personality has a very strong influence on their behavior. The second most important factor is the cognitive process, which shows that the way information is processed drives whether a person clicks on links; some people are more cautious, and some are a little more casual. The third most important factor is computer knowledge, which can effectively help people to better distinguish between phishing and legitimate emails.

The methods used in our study are interesting and can be applied in numerous practical applications to provide help in reducing people's susceptibility to phishing, and they can guide practitioners when investigating the susceptibility to phishing, detecting awareness, and implementing their training.

### 5.3. Implications

The main significance of this study is that it analyzes the variability of click-through rates between legitimate and phishing emails. Analyzing the factors influencing the susceptibility to phishing helps improve the effectiveness of resistance to 0-day attacks. The analysis of phishing emails allows for an effective corrective approach to reduce information leakage, significant property loss, and other damages that may be caused to the target.

Although phishing detection techniques are well developed, there are many people who are attacked by phishing. Many phishing attack techniques can bypass detection technologies because they do not produce attacks, and users cannot recognize them as attack software. Such attacks can have serious consequences when they are implemented. However, if we improve people's ability to defend against phishing and reduce susceptibility, we can effectively prevent and reduce losses.

We collected a large amount of data and reached certain conclusions through analysis, which further confirm and explore the influence of demographic, personality, cognitive processes, knowledge and experience, and security behavior factors on the susceptibility to phishing. For example, the correlation between personality and susceptibility to phishing was relatively high, with extraversion having the highest susceptibility among the personality traits that were analyzed. This is due to the manifestation of a more social and sexual preference.

Our study focuses on the initial steps of resisting to phishing that can identify potential phishing victims and understand their attributes and characteristics. These are predicted using a machine learning approach because machine learning approaches disregard the theory behind their application domains. Potential phishing victims are predicted using the model. However, because the data of the algorithm are easier to fit and the sample size of the data we collected is not large enough, further research is needed. Furthermore, we use static features, but very often, dynamic aspects are involved in phishing attacks, such as not seeing clearly and not noticing. Therefore, further research is needed.

In future research, the defense against phishing should be considered more thoroughly. Phishing is now one of the most threatening attacks in cyberspace security field. Although the current research on network defense technology has achieved some success, it remains severely threatened by APT and whale attacks. Therefore, preventing phishing should be one of the main priorities of an organization's information security awareness and training program.

## 6. Conclusions

The main purpose of this study was to evaluate the effectiveness of phishing emails, analyze the factors that influence phishing susceptibility, and predict potential phishing victims using the MPSPM model. We collected data on personal characteristics through recruited volunteers, tested the volunteers on phishing, and analyzed the acquired data using machine learning techniques. Our results show that identifiable models can predict potential phishing victims more accurately, and the MPSPM model can achieve a correct detection rate of 89.04% on the test set. Moreover, we analyzed the influencing factors behind phishing susceptibility, the most important of which are personality, cognitive processes, and computer knowledge. The computer knowledge characteristics are highly correlated with phishing, emphasizing that knowledge of network security is one of the key factors influencing the phishing susceptibility [11, 41].

## Figures and Tables

**Table 1:** Multidimensional attribute features.

Attribute	Features	Category	Frequency	Percentage
Demographics	Age	<20	67	6.06
		20–30	720	65.16
		30–40	107	9.68
		40–50	128	11.58
		>50	83	7.51
	Education level	Below high school	61	5.52
		Vocational high school/high school	109	9.86
		Undergraduates	610	55.2
		Graduate student or above	325	29.14
	Gender	Male	555	50.23
		Female	550	49.77
	Annual income	< ¥30,000	477	43.17
		¥30,000–¥100,000	369	33.39
		¥100,000–¥200,000	178	16.11
		> ¥ 200,000	81	7.33
Personality	Personality	Conscientiousness	124	11.22
		Extraversion	18	0.016
		Agreeableness	528	47.78
		Openness	443	40.09
		Neuroticism	42	0.038
Knowledge experience	Computer knowledge	High	250	22.62
		Middle	649	58.73
		Low	206	18.64
	Network security knowledge	High	179	16.19
		Middle	583	52.76
		Low	343	31.04
	Social engineering knowledge	High	129	11.67
		Middle	568	51.14
		Low	408	36.92
Susceptibility	Phished	Yes	609	55.12
		No	496	44.88

**Table 2:** Scores for each learning algorithm metric.

	Accuracy (%)	Precision (%)	Recall (%)	F1-score (%)
LR	77.52	80.55	77.12	78.80
DT	83.28	82.17	88.29	85.12
SVM	72.62	73.60	77.12	75.32
RF	84.14	83.75	87.76	85.71
GBDT	**89.04**	**87.87**	**92.55**	**90.15**
XGBoost	88.46	88.54	90.42	89.47
AdaBoost	88.47	87.75	91.48	89.58

Bold values represent the best performing values among all the modeled properties.

## Data Availability

The raw/processed data required to reproduce these findings cannot be shared at this time as the data also form a part of an ongoing study.
